# Endometriosis and aspirin: a systematic review

**DOI:** 10.3389/fendo.2024.1409469

**Published:** 2024-08-27

**Authors:** Yi Yang, HanHong Lai, ZhengJuan Li, Jun Zhang

**Affiliations:** ^1^ Department of Obstetrics and Gynecology, The Second Clinical Medical College, Jinan University, Shenzhen, China; ^2^ Center for Reproductive Medicine, Department of Obstetrics and Gynecology, Peking University, Beijing, China; ^3^ Department of Obstetrics and Gynecology, Shenzhen People’s Hospital (the Second Clinical Medical College of Jinan University and the First Affiliated Hospital of Southern University of Science and Technology), Shenzhen, China

**Keywords:** endometriosis, inflammation, aspirin, endometriosis-related symptoms, endometriosis treatments

## Abstract

**Introduction:**

Endometriosis is delineated as a benign yet steroid-dependent disorder characterized by the ectopic presence of endometrial glandular and stromal cells outside the uterine cavity, affecting estimated 10%–15% of women of reproductive age, 20%–50% of all women with infertility and costing a great economic burden per-patient. Endometriosis exerts pervasive influence on multiple facets of female reproductive physiology. Given its characterization as a chronic inflammatory disorder, escalated levels of pro-inflammatory cytokines were unequivocally recognized as well-established characteristics of endometriosis, which might attribute to mechanisms like retrograde menstruation, progesterone receptor resistance, and immune dysregulation. Therapeutic utilization of non-steroidal anti-inflammatory drugs (NSAIDs) like aspirin, analgesic agent for reducing pain, inflammation, and fever, could be holding promise in augmenting reproductive outcomes of endometriosis women. Therefore, the objective of this comprehensive review is to elucidate the intricate interplay between endometriosis and aspirin, both within the context of infertility and beyond. We meticulously explore potential pharmacological agents targeting endometriosis, which may concurrently optimize the efficacy of reproductive interventions, while also delving into the underlying mechanistic pathways linking endometriosis with inflammatory processes.

**Methods:**

We conducted a comprehensive search in the data available in *PubMed* and the *Web of Science* using the terms ‘endometriosis’ and ‘aspirin’. Then analyzed the identified articles based on established inclusion and exclusion criteria independently by three reviewers.

**Results:**

The survey of the chosen terms revealed 72 articles, only 10 of which were considered for review.

**Discussion:**

Based on the research available currently, it is not substantial enough to address the conclusion that aspirin shall be an effective therapeutic choice for endometriosis, further studies are needed to elucidate the efficacy, safety profile, and optimal dosing regimens of aspirin in the context of endometriosis treatment.

## Introduction

Endometriosis is widely recognized as a profound clinical challenge for women of reproductive age, profoundly compromising reproductive function across its entirety. Characterized by the aberrant presence of endometrial tissue beyond the confines of the uterine cavity, this condition afflicts approximately 10%-15% of reproductive-aged women and is identified in up to 50% of women facing infertility issues (1, 2). The typical clinical presentations of endometriosis include infertility, persistent pelvic pain, and escalating dysmenorrhea (3). The American Society for Reproductive Medicine initially formulated and subsequently revised its diagnostic criteria in 1997, categorizing endometriosis into three distinct subtypes: ovarian endometriomas (OMA), deep infiltration endometriosis (DIE), and superficial peritoneal lesions (SUP). Notably, the ectopic proliferation of endometrial tissue within the ovaries, either unilaterally or bilaterally, culminating in the formation of cystic masses referred to as OMA, predominantly impairs women’s reproductive capabilities by disrupting oocyte formation and maturation (4).

The etiological underpinnings of endometriosis remain elusive, although several theoretical frameworks have been posited to elucidate its pathogenesis (5). Foremost among these is the retrograde menstruation theory originally postulated by Sampson, which currently stands as the prevailing hypothesis in the field. The Sampson’s theory states that viable endometrial tissue refluxes into the peritoneal cavity through the fallopian tubes and subsequently implants into the peritoneal tissue and/or pelvic organs (6). Unfortunately, this theory fails to explain the fact that 80% to 90% of women had experienced retrograde menstruation but only 10% to 15% of women had endometriosis. Complementary theories include the epithelial–mesenchymal transition, hormonal dysregulation, immune system aberrations, and genetic predispositions are therefore posited as potential contributory factors to the onset and progression of endometriosis (7–10). Furthermore, an expanding body of literature underscores the increasingly recognized role of inflammation as a central contributor to the pathogenesis and persistence of endometriosis (11).

Treatment of endometriosis is aimed at suppressing lesion growth, alleviating pain, and ideally addressing the systemic effects of the disease. Surgical and pharmacological interventions are now two prevailing therapeutic strategies employed in the management of endometriosis. Surgical interventions, such as lesion ablation or excision, can offer temporary relief from symptoms, they shall also entail potential risks, notably the compromise of ovarian reserve, particularly due to thermal and electric injuries during ovarian cystectomy in cases with OMAs. For the majority of endometriosis patients, pharmacological treatments like progestogens or combined oral contraceptive pills (COCs) are commonly recommended (12). However, their effectiveness can sometimes be hindered by the inherent progesterone resistance observed in endometriotic lesions (13, 14). In addressing this therapeutic challenge, the use of gonadotropin-releasing hormone (GnRH) analogues, including both GnRH agonists and GnRH antagonists, has been advocated. Every medal has its reverse, the subsequent hypoestrogenic states can lead to discomforts such as vasomotor symptoms and decreased bone mineral density. Consequently, the use of “add-back therapy” of estrogen becomes necessary, which in essence exacerbates severity of endometriosis. In terms of relieving endometriosis-related pain, the concurrent use of analgesic agents, specifically NSAIDs, in conjunction with COCs or progestogens is supported by European Society of Human Reproduction and Embryology and the Royal College of Obstetricians and Gynecologists, and is recommended as the cornerstone pharmacotherapeutic approach for addressing indeterminate endometriosis-related pain.

Aspirin, as one of the three classic drugs in the history of medicine, chemically known as acetylsalicylic acid, possesses the capability to inhibit cyclooxygenase enzymes (COX-1 and COX-2), which are responsible for the conversion of arachidonic acid to prostaglandins, thereby reducing pain, inflammation, and fever. With an annual global consumption approximating 40,000 tons, aspirin represents a time-honored and frequently prescribed analgesic agent employed for pain management and inflammation attenuation (15). The use of aspirin for pain relief traces its roots back to ancient civilizations, with Chinese healers and Egyptians utilizing natural sources of salicylates for analgesic purposes. However, it wasn’t until the early 20th century, around 1900, that water-soluble aspirin tablets were first made available, marking a significant milestone in the discovery and development of this widely used medication (16). Since its inception, aspirin has evolved to become one of the most commonly prescribed analgesic and anti-inflammatory agents worldwide. Its versatile applications extend across various medical specialties, permeating virtually every department of medicine (17). From cardiology to oncology, and from rheumatology to gynecology, aspirin’s multifaceted pharmacological properties have established its indispensable role in contemporary medical practice.

Immunohistochemical investigations demonstrated that cyclooxygenase is expressed in various reproductive tissues, including the endometrial epithelium and fallopian tube secretory epithelial cells, although not in ciliated epithelial cells, cervical epithelium, and myometrial cells (18). As a pivotal enzyme regulating prostaglandin synthesis, COX-2 enhances invasiveness and promotes angiogenesis. Meanwhile, in ectopic endometrium of individuals with endometriosis, COX-2 expression is markedly upregulated, therefore facilitating the adhesion and invasiveness of endometrial cells (19–21).

The objective of this systematic review is to rigorously evaluate the current scientific literature regarding the potential association between aspirin and endometriosis, with an emphasis on inflammatory mechanisms. This includes an in-depth and critical analysis of both *in vivo* and *in vitro* experimental studies, and a comprehensive examination of clinical trial findings. The overarching objective is to elucidate potential therapeutic interventions that may hold promise for future clinical applications in the field of obstetrics and gynecology for the management of endometriosis.

## Methods

We searched the data available in *PubMed* and the *Web of Science*. The terms investigated include ‘endometriosis’ and ‘aspirin’. Three reviewers analyzed the data in an independent manner and only studies having at least one of the following characteristics were considered: observational or experimental, analytical or descriptive studies of the association between aspirin and endometriosis. Review and opinion studies were excluded as well as non-English manuscripts.

## Results

The survey of the chosen terms revealed 72 articles, only 10 of which were considered for review by satisfying the established inclusion criteria and fully analyzed.

In a seminal study by Nasiri N et al., a significant positive correlation was established between the expression levels of nuclear factor-κB (NF-κB) and the proliferative and adhesive capabilities of eutopic endometrial stromal cells. The NF-κB signaling pathway serves as a critical nexus within inflammatory cascades, dysregulation or aberrant activation of which has been implicated in the pathophysiology of a wide spectrum of inflammatory and autoimmune conditions, including endometriosis. Intriguingly, this observed relationship was found to be modifiable through interventions with aloe-emodin or aspirin (22). Contrarily, Massimi I et al. conducted an investigation revealing upregulated expression of multidrug resistance-associated protein 4(MRP4) mRNA and MRP4 protein in a peroxisome proliferator-activated receptor-alpha (PPAR-α) dependent manner (23).

In a comprehensive investigation conducted by Saad-Hossne R et al., the therapeutic efficacy of intralesional aspirin administration in mitigating endometriosis was rigorously assessed utilizing an established rabbit model of peritoneal endometriosis. Histological evaluations post-treatment revealed notable alterations within the endometriotic lesions, characterized by pronounced necrosis, hemorrhage, apoptosis, and fibrotic changes. Intriguingly, a substantial reduction in endometrial tissue foci, and in certain instances, complete eradication of endometrial tissue, was evident compared to the saline control cohort (24), Notably, these observations are congruent with earlier investigations by Siqueira JM et al (25). In an insightful study conducted by Efstathiou JA et al., utilizing a murine model, the differential impacts of various NSAIDs on the initiation and progression of endometriotic lesions were systematically evaluated. Comparative analysis revealed that among the NSAIDs investigated, celecoxib exerted the most pronounced reduction in lesion burden, followed by indomethacin, naproxen, sulindac, rofecoxib, and ibuprofen, respectively. Contrastingly, aspirin did not elicit any statistically significant impact on lesion burden compared to the control group (26) (**Table 1**).

**Table 1:** Previous *in vitro* and *in vivo* studies regarding endometriosis with aspirin.

Reference	Year	Objective	Study type	Significant findings
Nasiri N et al. (22)	2021	to assess whether aspirin and aloe-emodin as anti-inflammatory compounds could suppress the invasive activity of human endometrial stromal cells at stage IV endometriosis.	*In vitro* (Cellular)	Eutopic endometrial stromal cells seem to have a semi-invasive activity which is largely suppressed by aloe-emodin or aspirin (as potent NF-kB inhibitors).
Massimi I at al (23).	2018	to verify whether aspirin and other NSAIDs enhance MRP4 expression in 12Z human endometriotic epithelial cells and whether this was PPARa dependent.	*In vitro* (Cellular)	aspirin and other NSAIDs enhanced MRP4 mRNA and protein expression with PPARa expression induced
Saad-Hossne R et al. (24)	2016	to investigate the efficacy of intralesional 20% aspirin injection for treatment of experimental peritoneal endometriosis.	*In vivo* (rabbits)	Intralesional 20% aspirin injection caused total destruction of peritoneal endometriosis foci in rabbits.
Siqueira JM et al. (25)	2011	to estimate the effects of introduction of acetylsalicylic acid solution into peritoneal implants in autologous endometrium as a method for treating endometriosis.	*In vivo* (rabbits)	Acetylsalicylic acid solution effectively led to less growth of endometrial implants.
Efstathiou JA at al (26).	2005	To determine whether NSAIDs affect the establishment and progression of endometriotic lesions in a murine model.	*In vivo* (mice)	Chronic administration of certain NSAIDs could limit the progression of endometriosis in a murine model.
Ylikorkala O et al. (27)	1983	To study the production of prostacyclin (PGI2) and thromboxane A2 (TxA2) in endometriosis *in vitro*	*In vitro* (Cellular)	The production of prostanoids tended to be greater in the serosal than in the ovarian endometriosis.

In a meticulously designed double-blind, placebo-controlled clinical trial orchestrated by Ylikorkala O et al., a cohort of 18 patients afflicted with pelvic endometriosis was subjected to therapeutic regimens involving aspirin (acetylsalicylic acid), indomethacin, and tolfenamic acid. Surprisingly, the study outcomes diverged from anticipated results, revealing no substantial alleviation in endometriotic symptoms subsequent to NSAID administration, when compared to placebo treatments (27). Subsequently, in a similarly designed clinical trial by Kauppila A et al. in 1997, the impact of prostaglandin biosynthesis inhibitors, including aspirin, indomethacin, and tolfenamic acid, on endometriosis-associated symptoms was systematically assessed. Notably, the findings unveiled that tolfenamic acid exhibited heightened efficacy in mitigating endometriotic symptoms particularly during the menstrual phase. Conversely, both indomethacin and aspirin did not manifest any notable disparity in symptom relief compared to the placebo control group (28). In a pioneering pilot study published by Flannagan KS et al. in 2019, compelling evidence was presented for the first time suggesting that short-term therapy with aspirin and pravastatin, an HMG-CoA reductase inhibitor employed for lipid level reduction, could effectively attenuate high-sensitivity C-reactive protein levels. Furthermore, the combination of these pharmacological agents demonstrated promising potential in enhancing the outcomes of infertility treatments (29). Concurrently, Moon HS et al. conducted an exploratory pilot study to assess the effects of oral administration of piroxicam, at a dosage of 10 mg administered 1 to 3 hours prior to embryo transplant, on the *in vitro* fertilization-embryo transfer (IVF-ET) and frozen embryo transfer (FET) outcomes in patients presenting with endometriosis, tubal, and male infertility factors. Intriguingly, their findings elucidated that the piroxicam treatment group exhibited significantly elevated implantation and pregnancy rates compared to the control group. Specifically, the implantation and pregnancy rates were approximately doubled in patients with endometriosis factors following piroxicam treatment, as compared to the control (P<0.05) (30). In contrast, a study by Kumbasar S et al. presented divergent results. Their investigation encompassed 255 patients with primary or secondary infertility attributed to endometriosis, tubal, or male factors. The administration of either piroxicam (10 mg orally) or indomethacin (100 mg rectal suppository) prior to ET was assessed for its impact on implantation rates, miscarriage rates, and clinical pregnancy rates in patients undergoing IVF. Surprisingly, their data revealed that neither piroxicam nor indomethacin conferred any additional benefit in terms of implantation rates (P = 0.842), miscarriage rates (P = 0.964), or clinical pregnancy rates (P = 0.887) compared to the control group (31) (**Table 2**).

**Table 2:** Previous clinical trials of endometriosis with aspirin.

Reference	Year	Objectives	Design	Sample size	Significant findings
Ylikorkala O et al (27).	1983	To test the efficacy of three anti-prostaglandins in patients with pelvic endometriosis in addition to their *in vitro* data	A double-blind, placebo-controlled trial.	18 patients with pelvic endometriosis	Candidates sustained no relief for their endometriotic symptoms from the treatments with acetylsalicylic acid, indomethacin and tolfenamic acid
Kauppila A et al (28).	1979	To evaluate the effect of prostaglandin-inhibitors (acetylsalicylic acid, indomethacin and tolfenamic acid) on symptoms of endometriosis (especially pelvic pain)	A double-blind, placebo-controlled trial.	18 patients with pelvic endometriosis	Tolfenamic acid relieved endometriotic symptoms more effectively than placebo while indomethacin and acetylsalicylic acid did not differ from placebo during menstruation.
Moon, HS et al (30).	2004	To examine the effect of piroxicam treatment for priming of the uterus on the pregnancy outcome of IVF-embryo transfer (ET) programs.	Prospective, double-blinded placebo-controlled clinical study.	188 fresh and 78 frozen-thawed ET cycles of patients with endometriosis or other infertile factors	piroxicam increases implantation rate and pregnancy rate after IVF-ET in both fresh and frozen-thawed ET cycles. The beneficial effect seems to be more remarkable in patients less than 40 years old with tubal, male infertility, or endometriosis factors.
Kumbasar, Serkan et al (31).	2017	to evaluate the effect of NSAID administration before embryo transfer on pregnancy rates in women undergoing IVF/intracytoplasmic sperm injection(ICSI)-ET.	Prospective, double-blinded placebo-controlled clinical study.	255 patients diagnosed with primary or secondary infertility caused by endometriosis or other infertile factors	There is no additional effect on pregnancy outcome after NSAID administration before ET in patients undergoing IVF

## Discussion

With evolving theories attempting to elucidate its origins, the precise etiology of endometriosis remains elusive. Though Sampson’s theory positing that menstrual debris flows backward through the fallopian tubes, implanting and proliferating within the pelvic cavity holds sway and remains prevalent in contemporary discourse, the paradigm of inflammation has emerged as a pivotal determinant in the pathophysiology of endometriosis, and its role in the context of endometriosis has attracted significant attention and empirical validation. Inflammatory processes are now increasingly recognized as central to the initiation, progression, and perpetuation of endometriotic lesions. -

**Figure 1:**
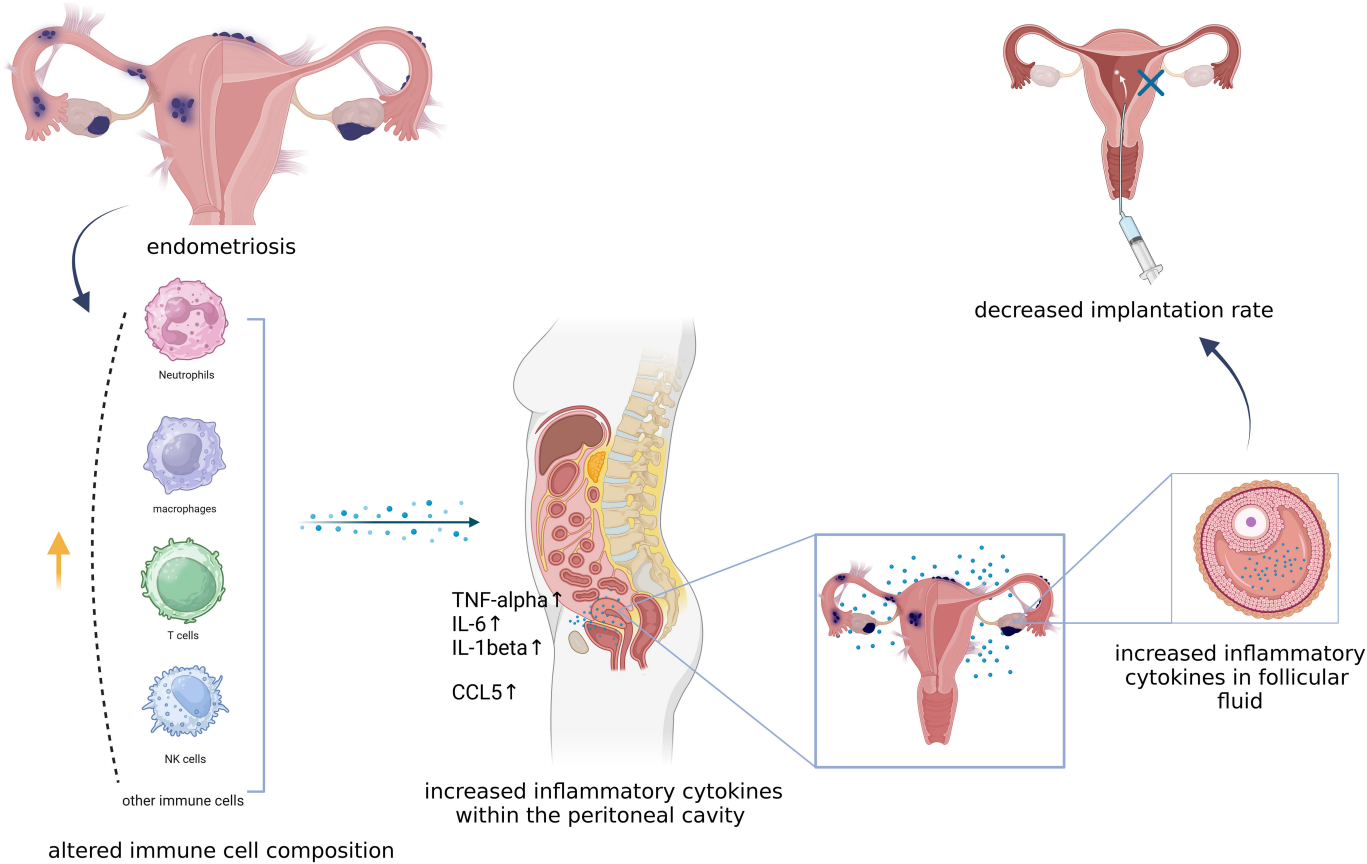
The relationship between immune cells and inflammation in endometriosis. this figure illustrates that neutrophils, macrophages, T cells and NK cells are all contributing to the pro-inflammatory environment both systematically and pelvically by secrecting TNF-alhpa, IL-6, IL-6beta as well as CCL5. Pro-inflammatory environment not only promotes the progression of endometriosis but also affects follicular fluid microenvironment and endometrial receptivity which might result in an unfavorable implantation. Created with BioRender.com.

Endometriosis is increasingly acknowledged not merely as a pelvic disorder but as a systemic condition (11, 32) with a dysregulated immune system and a proinflammatory environment (33). Neutrophils, pivotal granulocytes renowned for their role in combating bacterial invasions and preserving tissue integrity, have been implicated in the pathogenesis of endometriosis. This association is substantiated by strong evidence demonstrating heightened chemotactic activity in neutrophils derived from women with endometriosis compared to their counterparts in healthy control individuals. Concurrently, macrophages, serving as the vanguard of our primary immune defense, display increased activation of the proinflammatory transcription factor NF-κB and have been identified increased in the peritoneal fluid of women with endometriosis (34). However, while an elevation in macrophage numbers has been observed in the peritoneal fluid of women with endometriosis, these cells exhibit compromised phagocytic capabilities, culminating in inhibiting apoptosis and fostering proliferation of endometrial stromal cells, consistently, co-culture experiments have elucidated that macrophages can augment the proliferation and invasive potential of endometrial stromal cells (35, 36). Notably, estradiol, necessary for endometriosis development, has been identified as a crucial mediator of macrophage function in the context of endometriosis (35). Regulatory T cells (Tregs), a pivotal subset of T cells essential for modulating immune responses and sustaining maternal-fetal tolerance, elevated presence of which was detected in ectopic endometrial tissues and peritoneal fluid of endometriosis patients, suggesting that Tregs within the local microenvironment may contribute to the expansion and progression of endometriotic lesions (37, 38) (**Figure 1**).

The complex interplay of inflammatory mediators, cytokines, and immune cells orchestrates a pro-inflammatory microenvironment that fosters the adhesion, invasion, and survival of ectopic endometrial tissue outside the uterine cavity. Elevated levels of pro-inflammatory cytokines, such as interleukin-6 (IL-6), interleukin-1beta (IL-1β), tumor necrosis factor-alpha (TNF-α), and prostaglandin E2 (PGE2) have been consistently identified in women diagnosed with endometriosis (39, 40). PGE2 plays a crucial role in reproductive functions such as ovulation, implantation, parturition, and lactation. Elevated levels of prostaglandins can disrupt peritoneal function, leading to pain and interfering with essential processes like oocyte maturation, ovulation, and fertilization (41). These cytokines play pivotal roles in modulating inflammatory responses, immune cell activation, and tissue remodeling, thereby contributing to the pathophysiology of the disease. Additionally, the CCL5, also referred to as regulated upon activation normal T-cell expressed and secreted (RANTES), has been observed to be markedly increased in both endometrial tissues and follicular fluid of women with endometriosis. CCL5/RANTES functions as a chemoattractant for immune cells and has been implicated in the recruitment and activation of inflammatory cells at the ectopic endometrial sites, further perpetuating the inflammatory milieu characteristic of endometriosis (42–44). The gene encoding CCL5 receptor, known as CCR5, has already been proposed as a potential candidate gene for diagnosing endometriosis (45).

Abnormal high levels of COX-2 isoform and prostaglandins are presented in women suffering from endometriosis (46). Aspirin, however, targeting at cyclooxygenases therefore restricting the production of prostaglandins, has been testified in quite numbers of research to be effective in reducing endometrial lesion size and overall disease burden. Paradoxically, MRP4 expression, elevated of which may result in increased extracellular PGE2, has been found to be triggered in a PPAR-α dependent manner in endometrial cells treated with aspirin (23).

As it is the most commonly prescribed NSAID, aspirin ranks the highest among other NSAIDs in term of annual cost, for instance, Ibuprofen costs £0.86 for 24 tablets of 400 mg, leading to an annual expense of £40.05 (47). Low dose of aspirin, irreversibly targeting platelet cyclooxygenase, leading to decreased production of thromboxane A_2_, a potent platelet aggregator, is generally used to treat ischemic cardiomyopathy, atrial fibrillation, artificial heart valves, and arteriovenous fistulas in order to reduce the risk of cardiovascular events such as myocardial infarction and stroke. It also has carved out a notable niche in the realm of obstetrics and gynecology, particularly in the prevention of preeclampsia—a serious complication of pregnancy characterized by high blood pressure that might cause multiple organ damage and lead to both fetal and maternal mortality. Patients identified as being at high risk for developing preeclampsia are often prescribed oral low-dose aspirin, typically 50 to 100 mg daily, as a prophylactic measure to mitigate the risk and improve maternal and fetal outcomes (48, 49). Low-dose aspirin plays a pivotal role in the management of antiphospholipid antibody-associated recurrent spontaneous abortion, too (50), basing on antiplatelet properties of aspirin which is believed to help improve blood flow to the placenta and reduce the risk of miscarriage. In addition, aspirin also shows promise in improving the outcomes of IVF/ICSI procedures in women with endometriosis-associated infertility, as testified in previous clinical studies (30).

Over the past 15 years, research has documented the systemic effects of endometriosis. Women diagnosed with endometriosis are increasingly recognized as being predisposed to a higher risk of developing cardiovascular complications like stroke and coronary artery disease (51–53). The underlying pathophysiological mechanisms linking endometriosis to these cardiovascular conditions remain an area of active research but may involve chronic inflammation (53, 54). In addition to cardiovascular implications, endometriosis exerts a notable influence on metabolic processes, particularly within the liver and adipose tissue. This metabolic dysregulation can lead to alterations in body composition and energy metabolism. As a consequence, women with endometriosis often present with a lower body mass index (BMI) compared to individuals without (32, 55). Pain is the most debilitating and common symptom of endometriosis. Women diagnosed with endometriosis often experience cyclic and aggressing pelvic pain, typically during menstruation. Apart from acute pain during menstruation, women may also experience chronic pelvic pain, painful sexual intercourse, and pain associated with bowel and bladder functions. For many women, pain is persistent or chronic. _˄_Due to its rapid analgesic effects and lack of suppression on ovarian function, in comparison to hormonal treatment, aspirin demonstrates its own superiority, which may provide an alternative treating strategy.

However, as an antiplatelet drug, long-term use of aspirin could increase the risk of gastrointestinal or other major extracranial bleeds, too. This potential risk presents a limitation for the use of aspirin in the treatment of endometriosis. The current guidelines for occlusive vascular disease still largely recommended that in primary prevention, aspirin be used widely in patients at moderately raised of coronary heart disease, despite the possible bleeding risk (56). The management of bleeding disorders related to long-term use of aspirin involves a combination of strategies aimed at reducing bleeding risk (for example, prescribing the lowest effective dose of aspirin, using combination therapy with PPIs, etc.), monitoring for complications regularly (like a routinely endoscopy, regular follow-up of CBC, etc.), and treating any bleeding events that occur effectively.

Nevertheless, due to the lack of clinical data on long-term use of aspirin in endometriosis patients, the evidence for the effectiveness of NSAIDs in treating endometriosis was weak and contradictory, partly because of the vagueness regarding the exact analgesic used by individuals, for its handy accessibility (47). While there is ongoing research exploring the potential benefits of aspirin in managing endometriosis, its widespread adoption as a therapeutic agent for this condition has yet to be realized. Subsequently, in endometriosis patients, the long-term use of aspirin as primary prevention or therapeutic strategy should be evaluated, for example, assessing the risk of bleeding propensity relative to its preventive or therapeutic benefits. And more dedicated studies focusing on efficacy and safety of long-term use of aspirin in endometriosis patients are urgently needed. There are quite small number of research on the association between aspirin and endometriosis, so the correlation between aspirin and endometriosis remains elusive. More research is needed to explore the role of aspirin in the development and progression of endometriosis, aiming to identify new treatment targets and alternative medications for patients suffering from it.

The complex interplay of these pathological characteristics underscores the multifaceted nature of endometriosis and highlights the importance of targeting inflammatory pathways and cellular mechanisms in the development of novel therapeutic strategies for this debilitating condition (57). Both *in vitro* and *in vivo* studies have suggested that aspirin may exert curative effects on ectopic endometrial lesions by reducing cell infiltration and growth, but it is not substantial enough to state that aspirin shall be an effective therapeutic choice for endometriosis, and its long-term utilization necessitates careful evaluation, vigilant monitoring, and individualized patient management strategies to ensure both safety and therapeutic efficacy in clinical practice.

## Future directions

There is still need for further experimental research to elucidate the underlying mechanisms of action, optimal dosing regimens, and potential long-term effects of aspirin in endometriosis management. Moreover, there is also a pressing need for the design and execution of rigorously controlled and well-designed clinical trials to provide robust evidence regarding the efficacy, safety, and potential benefits of aspirin-based interventions in endometriosis patients.

## Conclusion

Based on the research available currently, it is not substantial enough to address the conclusion that aspirin shall be an effective therapeutic choice for endometriosis, although aspirin as an anti-inflammation drug targeting the COX enzymes, itself or its derivatives might help prevent/lower the risk of endometriosis development in the near future. Thus, further studies are needed to elucidate the efficacy, safety profile, and optimal dosing regimens of aspirin in the context of endometriosis treatment.

## Data Availability

The original contributions presented in the study are included in the article/supplementary material. Further inquiries can be directed to the corresponding author.
